# Decellularized nucleus pulposus matrix/chitosan hybrid hydrogel combined with nucleus pulposus stem cells and GDF5-loaded microspheres for intervertebral disc degeneration prevention

**DOI:** 10.1186/s10020-024-00777-z

**Published:** 2024-01-10

**Authors:** Tao Ma, Chen Liu, Quanlai Zhao, Yu Zhang, Liang Xiao

**Affiliations:** 1 Department of Hand and Foot Surgery, Yijishan Hospital of Wannan Medical College, No. 2 Zheshan West Road, Wuhu, Anhui 241001 China; 2 Department of Spine Surgery, Yijishan Hospital of Wannan Medical College, No. 2 Zheshan West Road, Wuhu, Anhui 241001 China; 3 Key Laboratory of Non-Coding RNA Transformation Research of Anhui Higher Education Institution, No. 2 Zheshan West Road, Wuhu, Anhui 241001 China; 4 Spine Research Center of Wannan Medical College, No.22 Wenchang West Road, Wuhu, 241001 China

**Keywords:** Intervertebral disc degeneration, NPSC, Decellularized nucleus pulposus matrix, Chitosan, Microspheres, GDF5

## Abstract

**Background:**

Intervertebral disc degeneration (IDD) is considered an important pathological basis for spinal degenerative diseases. Tissue engineering is a powerful therapeutic strategy that can effectively restore the normal biological properties of disc units. In this study, hydrogels loaded with growth/differentiation factor 5 (GDF5) and stem cells were combined to provide an effective strategy for nucleus pulposus regeneration.

**Methods:**

Nucleus pulposus stem cells (NPSCs) were obtained by low-density inoculation and culture, and their stem cell characteristics were verified by flow cytometry and a tri-lineage-induced differentiation experiment. A decellularized nucleus pulposus matrix (DNPM) and chitosan hybrid hydrogel was prepared, and GDF5-loaded poly(lactic-co-glycolic acid) (PLGA) microspheres were incorporated into the hydrogels to obtain a composite hydrogels with GDF5-loaded microspheres. Taking bone marrow mesenchymal stem cells (BMSCs) as a reference, the effect of composite hydrogels with GDF5-loaded microspheres on the chondrogenic differentiation of NPSCs was evaluated. A model of intervertebral disc degeneration induced by acupuncture on the tail of rats was constructed, and the repair effect of composite hydrogels with GDF5-loaded microspheres combined with NPSCs on IDD was observed.

**Results:**

Stem cell phenotype identification, stemness gene expression and tri-lineage-induced differentiation confirmed that NPSCs had characteristics similar to those of BMSCs. The rat DNPM and chitosan hybrid hydrogels had good mechanical properties, and the GDF5-loaded microspheres sustainably released GDF5. NPSCs grew normally in the composite hydrogels and gradually expressed a chondrocyte phenotype. Animal experiments showed that the composite hydrogels with GDF5-loaded microspheres combined with NPSCs effectively promoted nucleus pulposus regeneration and that the effect of the hydrogels on the repair of IDD was significantly better than that of BMSCs.

**Conclusion:**

GDF5-loaded microspheres combined with DNPM/chitosan composite hydrogels can effectively promote the differentiation of NPSCs into nucleus pulposus-like cells and effectively preventIDD.

## Background

Low back pain (LBP) is a common and frequently-occurring disease in modern society that seriously affects the quality of life of patients (Fritsch et al. 2023). There are many causes of LBP, including disc herniation, radicular pain, spinal stenosis, facet joint degeneration, and segmental spinal instability. These diseases often originate from the same pathological basis - intervertebral disc degeneration (IDD) (Lorio et al. 2023). At present, the etiology and pathophysiological mechanism of IDD are not completely clear, and the initiating factors of degeneration involve multiple aspects. For example, endplate cartilage is a key structural component of the intervertebral disc and is an important source of nutrition for intervertebral disc tissue. endplate cartilage degeneration is also an important cause of intervertebral disc degeneration, and maintenance of normal physiological function of the endplate cartilage is important for treating intervertebral disc degeneration (Malandrino et al. 2014; Farshad-Amacker et al. 2017). However, it is generally accepted that IDD begins in the nucleus pulposus, as evidenced by a decrease in the number of cells and a decrease in the content of proteoglycan (ACAN) and type II collagen (COL2A1), leading to a decrease in the water content of the nucleus pulposus, which in turn leads to changes in the properties of the disc tissue and its biomechanical properties, which in turn leads to changes in the properties of the intervertebral disc tissue and its biomechanical properties (Walker and Anderson 2004; Yurube et al. 2023).

The intervertebral disc is avascular tissue with very limited regeneration capacity, and once degeneration occurs, it is difficult to prevent or reverse. Current clinical treatments cannot fundamentally treat and prevent IDD and even accelerate the degeneration of the spine. The relatively ideal treatment for IDD is biological treatment that which promotes the synthesis of proteoglycans and other matrices, reverses the imbalance of matrix metabolism, delays or prevents the development of degeneration, and restores the biological function of the intervertebral disc (Borrelli and Buckley 2020). With the continuous development of molecular biology and tissue engineering technology, the use of tissue engineering to achieve cell transplantation to repair intervertebral discs has gradually become a hot spot of basic research in the field of spine surgery (Mercuri et al. 2011).

There are a variety of seed cells that can be used for cell transplantation in IDD, for example autologous nucleus pulposus cells, bone marrow mesenchymal stem cells (BMSCs) and induced pluripotent stem cells (IPSCs) (Shi et al. 2021; Kamatani et al. 2022). There are still a series of problems in the practical application of these cells: it is difficult to obtain materials, and the steps are cumbersome; furthermore, cells easily die after transplantation, and even the remaining cells will have greatly reduced activity, showing senescence and an abnormal cell phenotype (Soufi et al. 2023). Therefore, it is necessary to find an ideal source of seed cells for the treatment of IDD by cell transplantation. In addition, direct injection and transplantation of cells can easily lead to the leakage of cells from the injection site. Using a suitable scaffold as a carrier for cell transplantation can not only effectively prevent cell escape but also provide a suitable microenvironment for the colonization and differentiation of transplanted cells. In this study, a composite hydrogel system loaded with growth/differentiation factor 5 (GDF5) microspheres that can effectively control the release of GDF5 was developed. A series of experiments verified that the biomimetic system can induce the formation of nucleus pulposus-like cells in vivo and in vitro and effectively repair IDD.

## Materials and methods

### Nucleus pulposus stem cells (NPSCs) isolation and culture

A total of 200 Four-week-old Sprague Dawley rats were included in this study. The intervertebral discs were separated after rats sacrificed, and the surrounding muscles, ligaments and annulus fibrosus were removed to obtain jelly like nucleus pulposus tissue. The nucleus pulposus tissue was minced with ophthalmic scissors, collected into a centrifuge tube, digested with 0.25% type II collagenase (Gibco, USA) at 37 °C and 5% CO_2_ for 4 h, and centrifuged at 1500 rpm for 5 min, and the lower sediment was collected. Complete medium was added, and the cells were incubated in culture flasks. The culture medium was changed after 3 d, and the medium was changed every 2 d thereafter. After the cells were confluent to approximately 80%, 0.25% trypsin digestion (Gibco, USA) was performed to obtain P1-generation cells. The obtained P1-generation nucleus pulposus cells were counted with a chamber counting plate and seeded in a 10-cm culture dish at a density of 100 cells/cm^2^, and 6–8 ml of DMEM/F12 (Gibco, USA) medium containing 20% Fetal Bovine Serum (FBS, Gibco, USA) was added to each dish, after which the cells were cultured for 10 d at 37 °C and 5% CO_2_. The medium was changed every 3 d. The formation of cell colonies was observed under a light microscope, and colony-forming and increasing cell clusters (i.e., NPSCs) were obtained for subsequent experiments.

### BMSC isolation and culture

Four-week-old SD rats were sacrificed, the bilateral femurs and tibias were removed, and the muscles, periosteum and other tissues attached to the bones were removed. The two metaphyseal ends of the bones were cut with sterile scissors. The bone marrow cavity was rinsed with sterile phosphate buffered saline (PBS) solution until the rinse liquid was clear. The rinse liquid was divided into 50-ml centrifuge tubes for centrifugation and centrifuged at 1000 rpm for 5 min, and the supernatant was discarded. The cells were resuspended in low-glucose DMEM (Gibco, USA) containing 10% FBS (Gibco, USA) and inoculated into a 25-cm^2^ culture flask. Culture medium (4 ml) was added to the culture flask, and the cells were cultured at 37 °C and 5% CO_2_. The medium was first changed in 2–3 d, and then, the medium was changed every 3 d; the state of the cells was observed under a microscope and photographed. After the cells were 80% confluent, they were subcultured at a ratio of 1:3.

### Cell morphology assessment and proliferation assay

The morphology of the abovementioned human NPSCs and BMSCs was observed by fluorescence microscopy. NPSCs and BMSCs were seeded in 96-well plates at 5 × 10^2^, and their proliferation ability was quantitatively analyzed by the CCK-8 assay (Dojindo Molecular Technologies, Japan) after culturing for 0, 2, 4, 6, 8, 10 and 12 d.

### Tri-lineage-induced differentiation experiment

NPSCs and BMSCs were seeded in 24-well plates at a density of 2 × 10^4^ per well, and 1 ml of DMEM/F12 (Gibco, USA) medium containing 10% FBS (Gibco, USA) was added. After the cells reached 80% confluence, osteogenic differentiation induction medium (Gibco, USA), adipogenic differentiation induction medium (Gibco, USA) or chondrogenic differentiation induction medium (Gibco, USA) was added; the medium was changed every 3 d. After 21 d, the cells were fixed with 4% paraformaldehyde. Alizarin red staining solution was added to the cells in osteogenic induction medium, oil red O staining solution was added to the cells in adipogenic induction medium, and safranine O staining solution was added to cells in chondrogenic induction medium. The cells were observed under a microscope. Under the induction of differentiation media, stem cells will gradually differentiate into bone cells, adipocytes, and chondrocytes. Bone cells will be stained red with alizarin red, adipocytes will be stained orange with oil red O, and chondrocytes will be stained red with safranine O.

### Cell phenotyping

NPSCs and BMSCs were digested with 0.2% trypsin (Gibco, USA), collected in centrifuge tubes, and centrifuged at 1500 rpm for 5 min. The supernatant was discarded, and the cells were resuspended in PBS. The cells were counted and aliquoted into centrifuge tubes (1 × 10^6^ cells per tube). Antibodies (1:100, Santa Cruz, USA) against CD90, CD105, STRO1, CD45, CD34, or HLA-DR were added (1-2ul/tube). The cells were resuspended and incubated at room temperature for 20 min in the dark. Then, PBS was added, and the cells were centrifuged at 1500 rpm for 5 min, and the supernatant was discarded. After 3 repetitions, the expression of the above surface markers was detected by flow cytometry (BD Biosciences, USA).

### Realtime fluorescence quantitative PCR (qRT-PCR)

Total RNA was extracted from NPSCs and BMSCs using Trizol reagent (Gibco, USA). mRNA expression levels were assessed using a SYBR® Premix Ex TaqTM II kit (Takara, Japan). The cDNA amplification reaction conditions were as follows: pre-denaturation at 95 °C for 30 s; and denaturation at 95 °C for 5 s and annealing and extension at 60 °C for 25 s, for 50 cycles. The CT value of each sample was normalized to that for glyceraldehyde-3-phosphate dehydrogenase (GAPDH) as the housekeeping gene. The reaction for each pair of primers was replicated 3 times for each template, and the CT values obtained were averaged. The primer sequences are shown in Table 1.

**Table 1:** Primers and sequences used in this study

Gene	Primer	Primer sequence (5′→3′)
SOX-2	F	CCCCTGTGGTTACCTCTTCCTC
SOX-2	R	GGCCGCTCTGGTAGTGCTG
OCT4	F	GGCAAGCGACAAGCAGCGAC
OCT4	R	GGGAAAGGGACCGAGGAGTAC
NANOG	F	ACCCCGTTTCACTGTGTTAGC
NANOG	R	GACGGCAGCCAAGGTTATTAAA
ACAN	F	CATTCACCAGTGAGGACCTCGT
ACAN	R	TCACACTGCTCATAGCCTGCTTC
COL2A1	F	TGAGGGCGCGGTAGAGACCC
COL2A1	R	TGCACACAGCTGCCAGCCTC
GAPDH	F	GCTGAGAACGGGAAGCTTGT
GAPDH	R	GACTCCACGACGTACTCAGC

### Preparation of DNPM/chitosan composite hydrogel

Four-week-old SD rats were sacrificed to obtain jelly-like nucleus pulposus tissue under sterile conditions. The nucleus pulposus tissue was cut into approximately 1 mm^3^ pieces, placed in a mortar, and ground into powder after adding liquid nitrogen. The powdered nucleus pulposus tissue was placed in a constant temperature shaker at 37 °C, and 0.25% trypsin (Gibco, USA) was added for digestion for 24 h; the trypsin solution was replaced every 6 h. Subsequently, the samples were incubated in 10 mmol/L Tris-HCl containing 50 U/ml DNase and 1 U/ml RNase for 12 h. Then, 1% Triton X-100 was applied to the samples for 24 h, and the samples were washed 6 times with PBS. The precipitate was dissolved in 3% acetic acid, and 1.5 g of chitosan powder was dissolved in 100 ml of 3% acetic acid to prepare a chitosan solution. The hybrid hydrogel was prepared by mixing 4.5 ml 2.5% decellularized nucleus pulposus matrix and 1.5 ml 1.5% chitosan solution. The hybrid hydrogel was formed by adding 200 µl of the solution to a 24-well plate and then freeze drying for 24 h.

### Fourier transform infrared spectroscopy

Fourier transform infrared spectroscopy (Tanon, China) was used to detect and analyze the main components of the hybrid hydrogel. The infrared wavelength range was 600–4000 cm ^− 1^.

### Compression performance check

The hybrid hydrogels were tested in axial compression at room temperature using a universal material tester (Zwick/Roell, Germany). The hybrid hydrogels were placed on the equipment platform and compressed at a rate of 1 mm/min until rupture.

### Rheological property testing

Dynamic rheological properties were measured using a rheometer (Thermo Fisher, Massachusetts, USA) at 37 °C. The frequency was set to 1 Hz. The hydrogels were removed from the 24-well plate, and a dynamic strain scan from 0.1 to 100 Hz was performed at room temperature to test the storage modulus (G’) and loss modulus (G’’) over time.

### Preparation of composite hydrogel with GDF5-loaded microspheres

GDF5-loaded PLGA microspheres were prepared using a water-oil emulsion solvent evaporation method. PLGA (150 mg, Birmingham Polymers, USA) was dissolved in 2 ml of dichloromethane, after which GDF5 (50 µg/ml) was added. The mixture was then placed in an ice bath and emulsified using a homogenizer (8000 rpm, 30 s). Next, 50 ml of polyvinyl alcohol solution was added to the emulsion, followed by homogenization at 4000 rpm for 2 min. Then, 50 ml of deionized water was added to the obtained product, and dichloromethane was evaporated by magnetic stirring for more than 3 h. Finally, PLGA microspheres were collected by centrifugation at 8000 rpm for 10 min, washed three times with deionized water to ensure the removal of free non-sphered components, lyophilized and stored at -20 °C for later use. After the preparation of microsphere, centrifuge the supernatant and use ultraviolet spectrophotometer to test the concentration of dissociative GDF5. Calculate the encapsulation efficiency of GDF5 using the following formula: The content of GDF5 encapsulated in microsphere/the theoretical content of GDF5 encapsulated in microsphere×100%. Microsphere particle size was measured using a laser scattering particle size analyzer. Under sterile conditions, the GDF5-loaded microspheres were uniformly mixed with the DNPM/chitosan hybrid hydrogels, and the test tube was inverted. If the solution did not flow within 30 s, the sample solution was considered to have transformed into a gel.

### Scanning electron microscopy (SEM)

The morphologies of the hybrid hydrogels and PLGA microspheres were observed by SEM (Hitachi, Japan). The hybrid hydrogels and PLGA microsphere samples were sputter-coated with gold and observed by SEM at an accelerating voltage of 3 kV.

### In vitro release assay of composite hydrogel with GDF5-loaded microspheres

The composite hydrogels with GDF5-loaded microsphere samples were placed in a 5-ml centrifuge tube, after which 2 ml of PBS solution was added and the samples were placed on a constant temperature shaker at 37 °C and continuously shaken at 150 rpm. The supernatant was aspirated at 0 d, 3 d, 6 d, 9 d, 12 d, 15 d, 18 d, 21 d, 24 d, 27 d, and 30 d. The GDF5 content in the supernatant was assessed using the Bicinchoninic acid (BCA) method, and a cumulative release curve was drawn.

### In vitro degradation assay of composite hydrogel with GDF5-loaded microspheres

The composite hydrogels were completely immersed in PBS solution, and after reaching swelling equilibrium at 0 d, 3 d, 6 d, 9 d, 12 d, 15 d, 18 d, 21 d, and 24 d, the gels were removed and washed with ultrapure water. After freeze-drying the hydrogels, the weight was measured and the remaining weight (%) was calculated as follows: remaining weight = Wt/W0 × 100%, where W0 represents the weight before degradation, and Wt represents the weight after degradation at different time points.

### Preparation of hydrogel-conditioned medium

The two composite hydrogels loaded with GDF5 microspheres and those without GDF5 microspheres were washed repeatedly with PBS buffer containing 10% penicillin and streptomycin (Gibco, USA) and placed in a 6-well plate, and 5 ml of serum-free medium was added to each well, followed by incubation in a 5% CO_2_ incubator at 37 °C. The medium was extracted after 3 d, 6 d, 9 d, 12 d, 15 d, 18 d, and 21 d of incubation. The extracted medium was centrifuged at 1000 rpm for 10 min, the supernatant was collected, and the bacteria were filtered. Then, 10% FBS (Gibco, USA) and 1% penicillin-streptomycin solution (Gibco, USA) were added, and the samples were transferred into 50-ml sterile centrifuge tubes for stem cell culture.

### Effects of hydrogel-conditioned medium on stem cell proliferation

NPSCs and BMSCs were seeded in 96-well plates at 5 × 10^2^, and the corresponding four hydrogel-conditioned mediums were replaced per the set medium exchange time. After culturing for 0, 2, 4, 6, 8, 10, and 12 d, the proliferative capacities of NPSCs and BMSCs were quantitatively analyzed by the CCK-8 assay (Dojindo Molecular Technologies, Japan).

### Effects of hydrogel-conditioned medium on the chondrogenic differentiation of stem cells

NPSCs and BMSCs were seeded into 6-well plates at a density of 1 × 10^6^ cells/well and induced by adding chondrogenic medium (Gibco, USA). After the medium was changed per the set time points, the cells were collected on the 14th and 21st days. The expression levels of the cartilage marker molecules ACAN and COL2A1 were assessed by RT‒qPCR and Western blot.

### 3D observation of stem cells in composite hydrogel

An appropriate amount of FITC-containing freeze-dried hydrogel scaffold with microspheres was blended with 1 × 10^6^/ml NPSC, and a cell hydrogel scaffold colloid formed after mixing evenly. Next, 3 ml/well cell hydrogel scaffold colloid was added to a round-bottomed confocal petri dish, an appropriate amount of medium containing 10% serum was added, and the dish was placed in an incubator for 24 h. After fixation with formalin for 20 min, DiDiethylethoxysilane (DID) fluorescent dye was added to stain the cell membrane for 20 min. After washing the dishes with PBS for 9 min, the distribution of stem cells and microspheres in the hydrogels was observed by 3D confocal microscopy (Leica, Germany).

### Observation of stem cell morphology in composite hydrogel

The freeze-dried composite hydrogel scaffolds were mixed with 1 × 10^7^/ml BMSCs or NPSCs; the mixture was added to 24-well plates (1 ml/well) and then cultured in an incubator after adding an appropriate amount of medium containing 10% serum. The plates were removed at set time points for SEM observation, and cytoskeleton formation was assessed by phalloidin staining (Gibco, USA).

### Live and dead cell staining of stem cells in composite hydrogel

The following four groups were analyzed: composite hydrogels + BMSCs (composite hydrogels, CH + BMSCs), composite hydrogels + NPSC (CH + NPSCs), composite hydrogels with GDF5-loaded microspheres + BMSC (GDF5/CH + BMSCs) and composite hydrogels with GDF5-loaded microspheres + NPSCs (GDF5/CH + NPSCs). The cells were removed at the set time points for staining using a live-dead cell staining kit (Gibco, USA); that is, a green pyridine iodide (PI) fluorescent dye (Ex/Em = 488/518 nm) that penetrates cell membranes and a red rhodamine dye (Ex/Em = 488/615) that does not penetrate cell membranes were used to stain live and dead cells and evaluate cell growth.

### Detection of cartilage marker expression in stem cells in composite hydrogel

For the four groups described above, the hydrogels were removed at the set time points, fixed with formalin, embedded and solidified, sliced, and stained with COL2A1 fluorescent antibody (1:1000, Abcam, USA), and the expression of corresponding proteins on the cell surface were observed by fluorescence microscopy.

### Evaluation of the in vivo repair of IDD by composite hydrogel with GDF5-loaded microspheres

After being approved by the animal experiment committee of the institution, 48 male SD rats aged 8 weeks were randomly divided into a control group (Ctrl), operation group (surgery), and composite hydrogel groups (CH + BMSCs, CH + NPSCs, GDF5/CH + BMSCs, and GDF5/CH + NPSCs), with 8 rats in each group. Ctrl rats were not subjected to any surgical manipulation. SD rats in the operation group, CH + BMSC group, CH + NPSC group, GDF5/CH + BMSC group and GDF5/CH + NPSC group were anesthetized with pentobarbital (40–50 mg/kg) and placed in a supine position, and their limbs were immobilized. The abdomen was cleaned after shaving the hair from the skin, and draping was completed after the area was sterilized with iodophor. Under X-ray fluoroscopy, a 21G micropuncture needle (Hamilton, Bonaduz, Switzerland) was used to puncture the caudal intervertebral disc to the central nucleus pulposus. After rotating the needle 360°, the puncture needle was removed, the puncture point was disinfected with iodophor, and 50,000 units of penicillin was administered intramuscularly to prevent infection. In addition to the abovementioned surgical operations, the rats in the CH + BMSC group, CH + NPSC group, GDF5/CH + BMSC group and GDF5/CH + NPSC group were injected with different compound hydrogels in the intervertebral space using a 33 G micropuncture needle. Iodophor disinfection was also performed, and 50,000 units of penicillin was administered intramuscularly to prevent infection. The above 6 groups of rats underwent plain magnetic resonance imaging (MRI) of the lumbar spine 2 months after puncture modeling to assess intervertebral disc degeneration; the Pfirrmann grading method was used to evaluate each rat and compare groups. All rats were sacrificed to obtain rat tail intervertebral disc tissue samples, and safranin fast green staining was used to observe the morphological changes in the intervertebral discs in each group. Immunohistochemistry was used to assess the differences in COL2A1 expression in the nucleus pulposus tissue of the surgical segment of the rats in each group.

## Result

### Characteristics of NPSCs and BMSCs

**Fig. 1:**
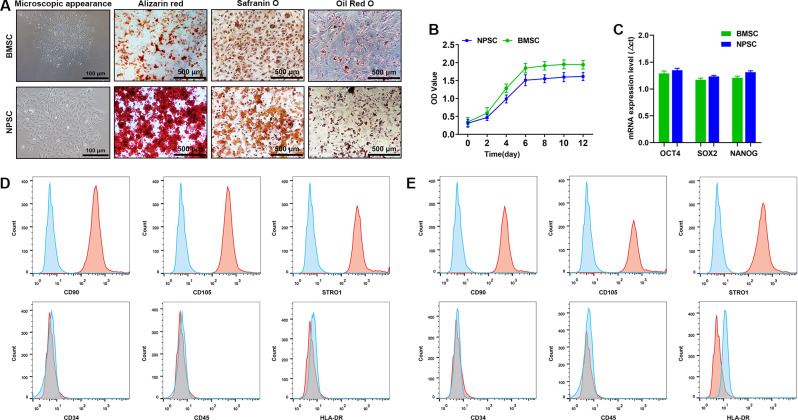
NPSCs had characteristics similar to those of BMSCs. (**A**) NPSCs had three lineage differentiation abilities. (**B**) There was no significant difference in the proliferation ability between NPSCs and BMSCs. (**C**) qPCR was used to detect the expression levels of phenotypic genes of stem cells. (**D–E**) Flow cytometry was used to detect the stem cell immunophenotype expression of NPSC and BMSC. (n ≥ 3)

Both NPSCs and BMSCs grew in colonies when inoculated at low density. The former formed sunflower-like colonies, and the latter formed swirl-like colonies; both exhibited multidirectional differentiation (Fig. 1A). The CCK-8 assay showed that the two groups of cells had a similar “S”-shaped growth trend and that there was no significant difference in the proliferation ability between the two groups (Fig. 1B). Quantitative PCR indicated that both NPSCs and BMSCs expressed stem cell-specific genes, i.e., OCT-4, SOX-2 and NANOG, with comparable expression levels (Fig. 1C). In addition, flow cytometry showed that BMSCs (Fig. 1D) and NPSCs (Fig. 1E) were positive for many stem cell markers, such as CD90, CD105, and STRO1, but negative for the hematopoietic stem cell marker CD34, pan-hematopoietic stem cell marker CD45, and leukocyte marker HLA-DR.

### Characterization and detection of DNPM/chitosan hybrid hydrogel

**Fig. 2:**
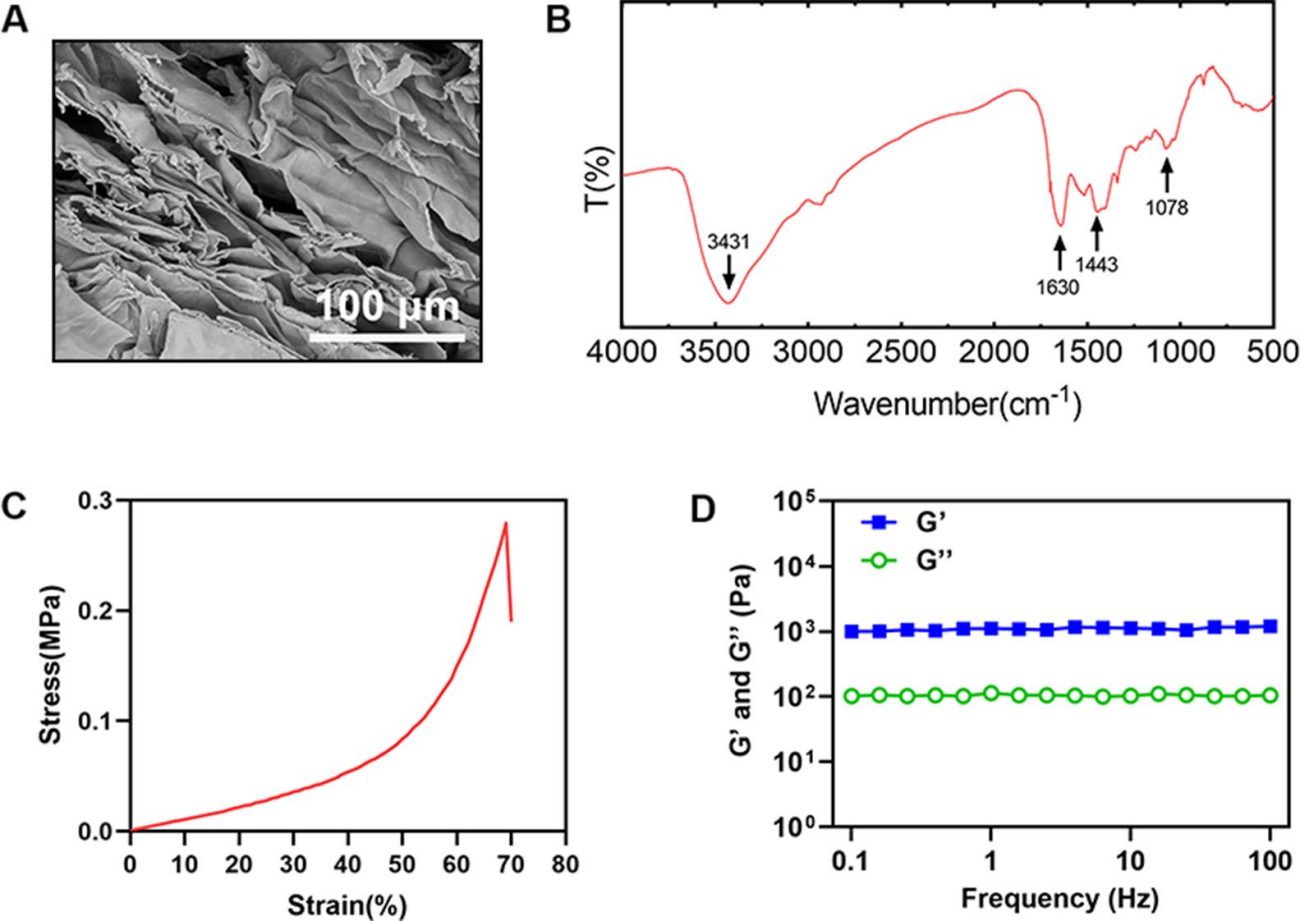
The composite hydrogels had good mechanical properties. (**A**) SEM image of DNPM/chitosan composite hydrogel. (**B**) Fourier transform infrared spectroscopy was used to analyze the main components of the composite hydrogel. (**C**) The composite hydrogel was tested for compression performance check. (**D**) The composite hydrogel was tested for rheological property. (n ≥ 3)

Under SEM, the composite hydrogel had a smooth porous network structure with good connectivity between pores (Fig. 2A). The most prominent feature on the FTIR spectrum is the absorption peak. The absorption peak represents the frequency at which a substance absorbs infrared light. The number and intensity of absorption peaks can be used to determine the functional groups of a substance. In this study, FTIR showed that there was amide A absorption peak at 3431 cm^− 1^, which was related to the stretching vibration of N-H and O-H bonds. There was amide I absorption peak at 1630 cm^− 1^, which was related to the stretching vibration of C = O. There was amide II absorption peak at 1443 cm^− 1^, which was related to N-H bond bending vibration and C-N bond stretching vibration. There was absorption peak at 1078 cm^− 1^, which was related to the stretching vibration of C-O-C bond. Due to the fact that collagen was mainly composed of amides I, II, and III, and 1078 cm^− 1^ was also in the sugar band, FTIR indicated that DNPM was mainly composed of collagen and polysaccharides (Fig. 2B). Compression test results indicated that with increasing compressive strain, the compressive stress of the composite hydrogel increased and that the composite hydrogel failed when the compression was close to 70%, indicating good elasticity and suitability for stem cell differentiation and in vivo tissue application (Fig. 2C). Regarding the rheological test results, the composite hydrogel had a stable storage modulus (G′) that was much higher than the loss modulus (G′′), suggesting that the composite hydrogel had good elasticity (Fig. 2D). The PH of the hydrogel was between 7.1 and 7.3, close to neutral, and the cells can grow and survive in the gel.

### Characterization and assessment of composite hydrogel with GDF5-loaded microspheres

**Fig. 3:**
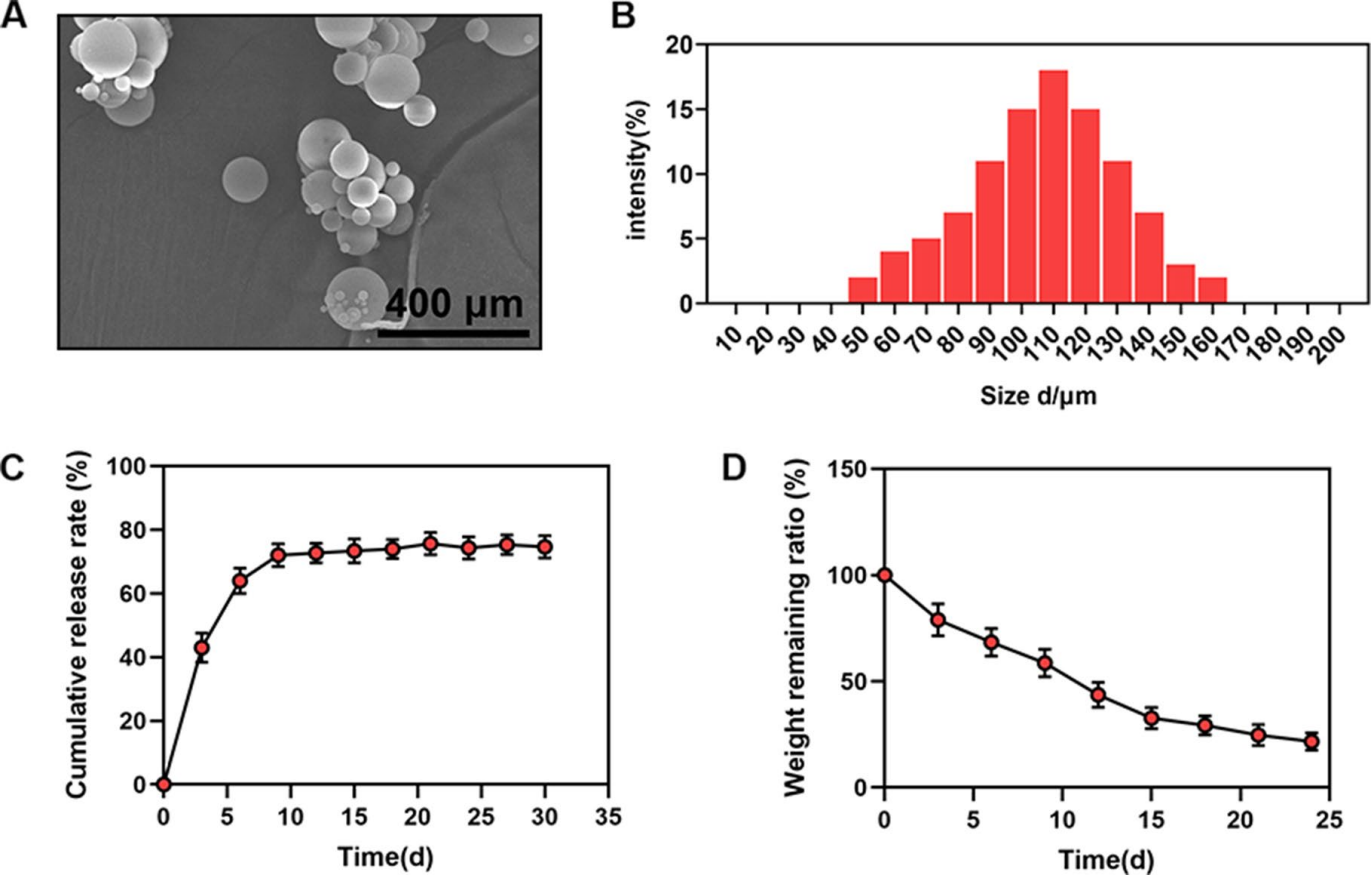
GDF-5 can be slowly released into the composite hydrogel. (**A**) SEM image of GDF5-loaded microspheres. (**B**) Measurement of microsphere diameter. (**C**) Release detection of GDF-5 in vitro. (**D**) Degradation detection of GDF5-loaded microspheres in vitro. (n ≥ 3)

SEM showed that the GDF5 microspheres had a uniform spherical shape with a particle size range of 50–160 μm (average, 110 μm). The encapsulation efficiency is 75.1%. (Fig. 3A, B). To examine the release efficiency of GDF5 from PLGA microspheres, a 30-day continuous test was conducted with the release solution of composite hydrogel with GDF5-loaded microspheres. GDF5 showed a slow release trend, and the release efficiency reached a plateau around the 10th day (Fig. 3C). In addition, the degradation experiments showed that the residual mass of the composite hydrogels exhibited a gradual decreasing trend, decreasing to 20% of the original mass after 24 d of culture.

### Effects of composite hydrogel with GDF5-loaded microspheres on the chondrogenic differentiation of NPSCs and BMSCs

**Fig. 4:**
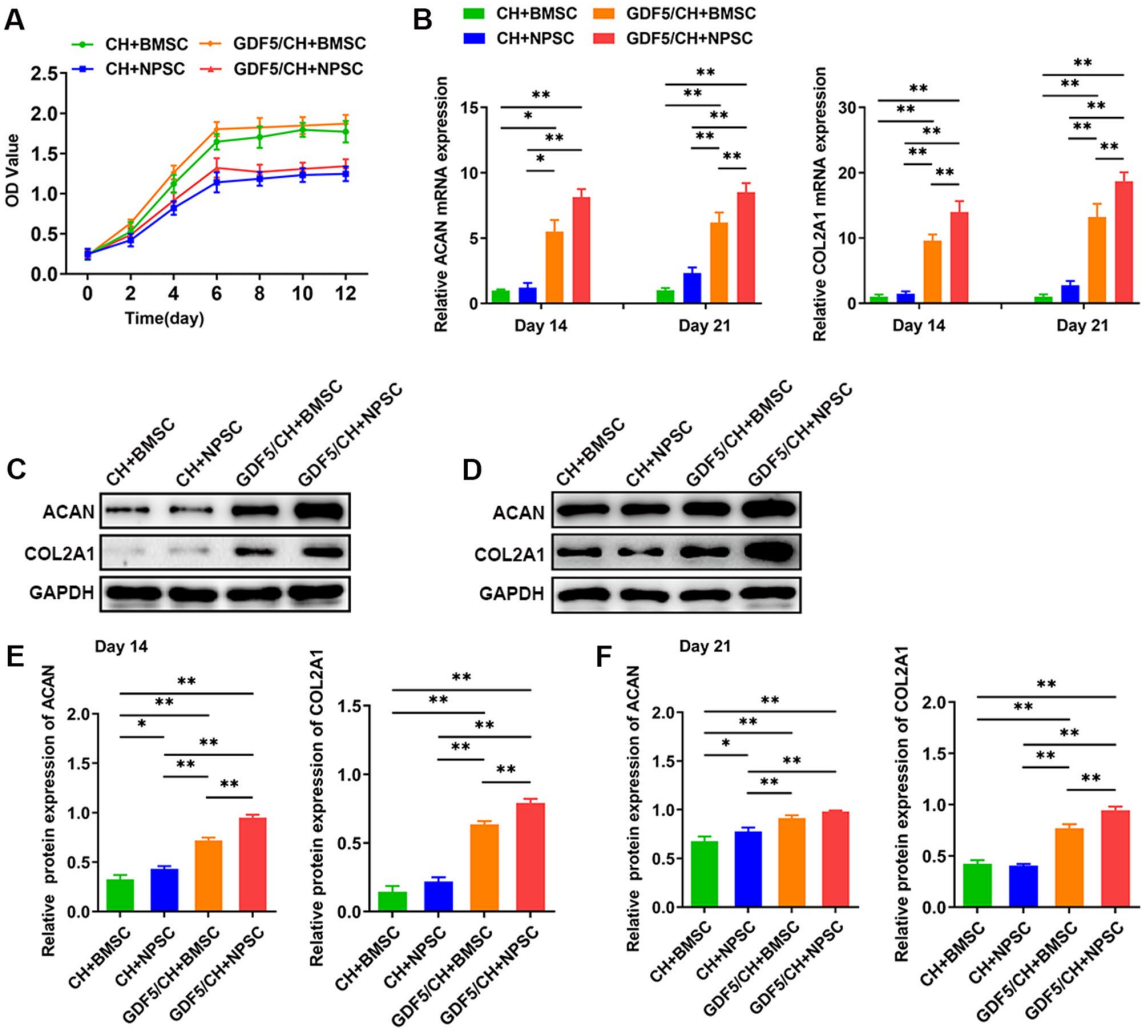
The composite hydrogels with GDF5-loaded microspheres significantly increased the chondrogenic level of NPSCs. (**A**) CCK-8 assay was used to detect the proliferation level of NPSCs and BMSCs under the four hydrogel-conditioned mediums at different time points. (**B–E**) RT-qPCR and western blot were used to detect the expression levels of phenotypic genes and proteins of NPSCs and BMSCs under the four hydrogel-conditioned mediums at 14 and 21 d. (n ≥ 3, *p < 0.05, **p < 0.01)

When using two types of hydrogel medium to induce the chondrogenic differentiation of NPSCs and BMSCs, the cell proliferation abilities of the CH + BMSC group, CH + NPSC group, GDF5/CH + BMSC group and GDF5/CH + NPSC group were significantly different (Fig. 4A). However, the expression levels of the cartilage markers ACAN and COL2A1 in cells of the GDF5/CH + BMSC group and GDF5/CH + NPSC group were significantly higher than those in cells of the CH + BMSC group and CH + NPSC group; cells in the GDF5/CH + NPSC group had the highest expression level (Fig. 4B-E).

### Compatibility of composite hydrogel with GDF5-loaded microspheres with NPSCs and BMSCs

**Fig. 5:**
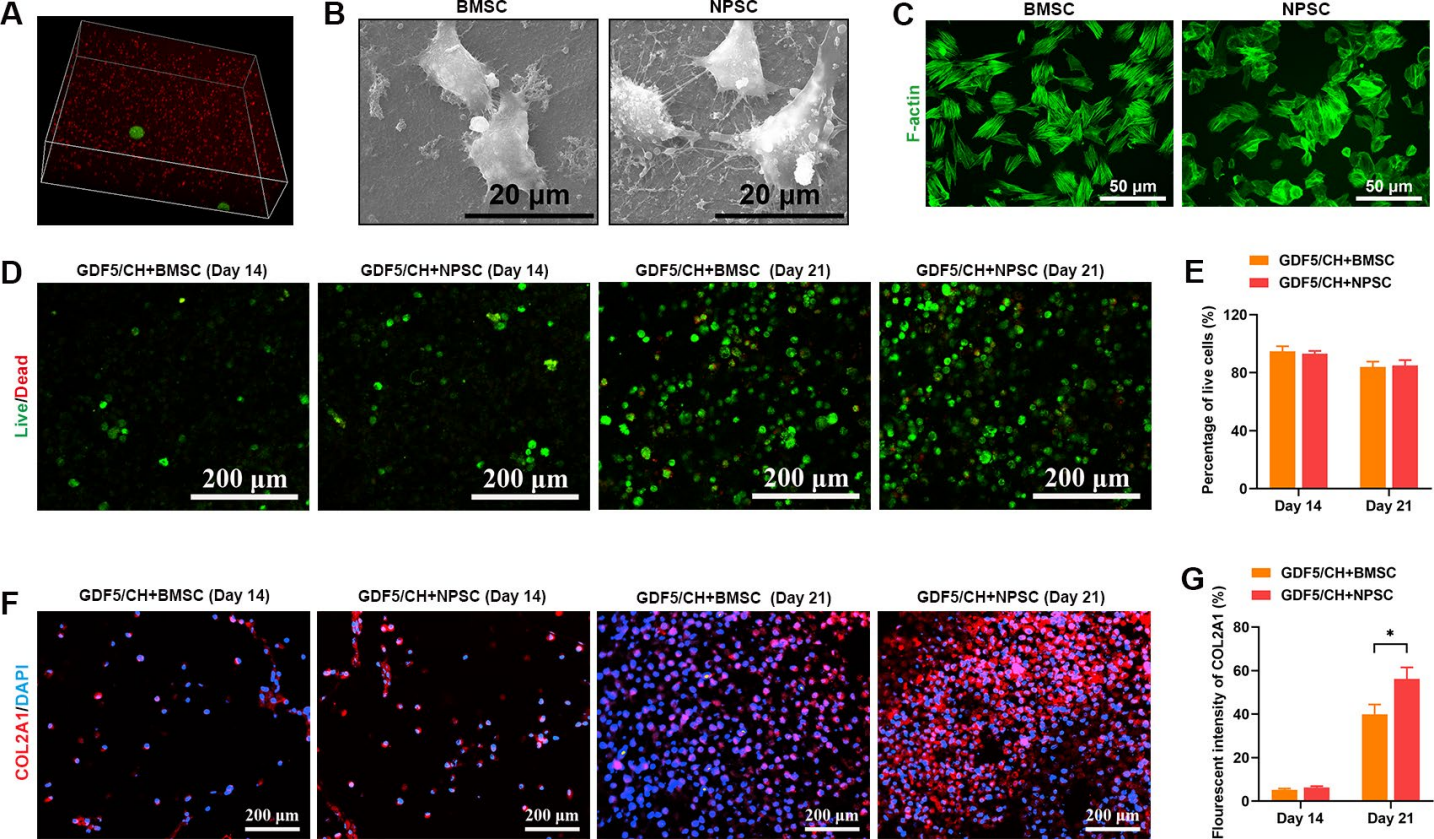
The composite hydrogels with GDF5-loaded microspheres can promote NPSCs to secrete more extracellular matrix. (**A**) 3D observation of stem cells and microspheres in composite hydrogel. (**B**) Observation of NPSCs and BMSCs adhesion in composite hydrogel by SEM. (**C**) Phalloidin staining was used to observe the skeleton spreading of NPSCs and BMSCs in composite hydrogel. (**D–E**) Live-dead cell staining was used to evaluate the growth of NPSCs and BMSCs under the four hydrogel-conditioned mediums at 14 and 21 d. (**F–G**) Immunofluorescence was used to detect the COL2A1expression level of NPSCs and BMSCs under the four hydrogel-conditioned mediums at 14 and 21 d. (n ≥ 3, *p < 0.05)

3D observation showed that the stem cells in the composite hydrogel were uniformly distributed, and the microspheres were embedded in the hydrogel, surrounded by many stem cells (Fig. 5A). SEM and cytoskeleton staining showed that NPSCs and BMSCs well adhered and spread in the composite hydrogel (Fig. 5B, C). Live and dead cell staining showed that NPSCs and BMSCs grew well in the hydrogel; there was no significant difference in the percentage of viable cells between the two cell types at 14 and 21 d of culture (Fig. 5D, E). In addition, fluorescence staining showed that there was no significant difference in the content of COL2A1 protein secreted by NPSCs and BMSCs at 14 d of culture but that the COL2A1 protein content secreted by NPSCs was significantly higher than that secreted by BMSCs at 21 d of culture (Fig. 5F, G).

### Repair effect of composite hydrogel with GDF5-loaded microspheres on IDD

**Fig. 6:**
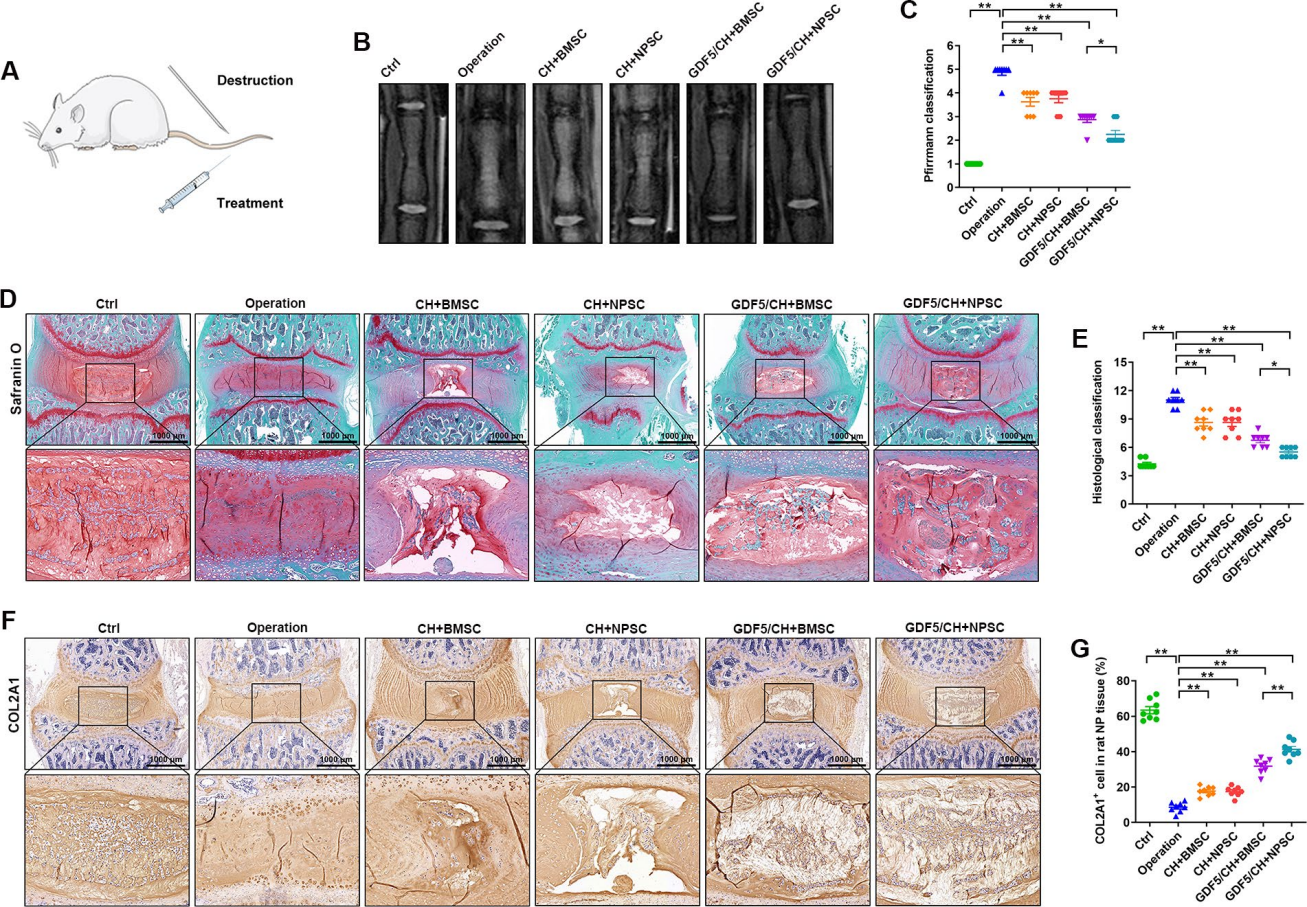
The composite hydrogels with GDF5-loaded microspheres loaded with NPSCs had the best repair effect on IDD. (**A**) Description of animal experiment. (**B–C**) Observation of rat tail intervertebral disc degeneration in each group by MRI and classification analysis. (**D–E**) Observation of rat tail intervertebral disc in each group by safranine and fast green staining and classification analysis. (**F–G**) The COL2A1 expression level in NP tissues in the six groups were determined by immunohistochemistry and quantitative analysis. (n ≥ 3, *p < 0.05, **p < 0.01)

A tail puncture-induced IDD rat model was constructed to verify whether and to what extent the composite hydrogel with GDF5-loaded microspheres had a prevent effect (Fig. 6A). Two months after modeling, MRI indicated that in the Ctrl group, the signal for the intervertebral disc was normal and that in the surgery group, the signal for the intervertebral disc was significantly reduced due to puncture damage, the intervertebral disc height exhibited significant loss, and the degree of degeneration was most severe. Compared with that in the operation group, the signal strength of IDD in the CH + BMSC group, CH + NPSC group, GDF5/CH + BMSC group and GDF5/CH + NPSC group was significantly increased; the GDF5/CH + NPSC group had the strongest signal strength (Fig. 6B, C). The trend for the degree of IDD in the surgical segment, as observed by safranin fast green staining, was consistent with the MRI results; that is, the GDF5/CH + NPSC group had the mildest degree of IDD in the surgical segment (Fig. 6D, E). Finally, the immunohistochemical results also showed a significant decrease in COL2A1 protein expression in the nucleus pulposus of the operation group due to puncture damage in the surgical segment. The expression of COL2A1 in the nucleus pulposus of the CH + BMSC group, CH + NPSC group, GDF5/CH + BMSC group, and GDF5/CH + NPSC group was significantly higher than that of the operation group, with the highest expression observed in the GDF5/CH + NPSC group. (Fig. 6F, G).

### Statistical analysis

We used SPSS 18.0 for statistical analysis. One-way ANOVA was used for accessing differences among groups. Comparison between two groups was accessed by least square difference (LSD) method. The mean difference is expressed as a 95% confidence interval. Values of *P* < 0.05 were considered statistically significant.

## Discussion

Clinically, for IDD diseases, both surgical treatment and conservative treatment can only temporarily relieve symptoms and cannot fundamentally resolve the pathological state of IDD (Fontana et al. 2015). Tissue engineering treatment with the purpose of delaying or even reversing IDD has gradually attracted much attention. Seed cells, scaffolds and cytokines are the three main factors for tissue engineering repair. Previous studies have shown that endogenous stem cells exist in almost all adult tissues and organs and that these stem cells have strong potential in repair, regeneration and homeostasis maintenance after tissue injury (Zhou et al. 2023). An increasing number of studies have also confirmed the presence of stem cells in intervertebral disc tissue; these cells originate from different anatomical regions of the intervertebral disc, such as endplate cartilage, the annulus fibrosus, and the nucleus pulposus (Zhang et al. 2021; Luo et al. 2022). In this study, NPSCs were successfully extracted from rat nucleus pulposus tissue by low-density inoculation and culture. Stem cell phenotype identification, stemness gene expression and tri-lineage differentiation induction confirmed that NPSCs had characteristics similar to those of BMSCs. The above studies show that NPSCs, similar to BMSCs, have potential as ideal seed cells for cell therapy and tissue engineering. However, regulating the directed differentiation of exogenously transplanted stem cells into nucleus pulposus-like cells and performing repair functions are urgent issues that need to be addressed.

Tissue engineering scaffolds are carriers of seed cells. An ideal scaffold must be conducive to cell attachment and proliferation, have good biocompatibility, absorbability and degradability, can reshape the extracellular matrix environment and promote the specific differentiation of seed cells (Choy and Chan 2015). At present, tissue engineering scaffolds include artificial polymer materials, natural biological materials and composite materials. Compared with other materials, the advantages of natural biomaterials lie in their excellent biocompatibility, ability to interact with cells and degradability, allowing them to be widely used in tissue engineering and especially suitable for the construction of tissue-engineered nucleus pulposus. In addition, chitosan is the only natural cationic alkaline polysaccharide in nature, and its cationic properties allow it to capture high-valent anionic proteoglycans produced by intervertebral disc cells, which is beneficial to the maintenance of intervertebral disc matrix function (Du et al. 2022). Roughley et al. (Roughley et al. 2006) planted nucleus pulposus cells in chitosan gel material and found that the cells could better maintain their phenotype and synthesize a large amount of extracellular matrix, such as proteoglycans. Richardson et al. (Richardson et al. 2008) cocultured BMSCs with chitosan/sodium glycerophosphate hydrogels, and the results showed that the hydrogel scaffold promoted the differentiation of BMSCs into nucleus pulposus cells. In this study, rat nucleus pulposus tissue was chosen as the raw material for the scaffold, nucleus pulposus cells in the tissue were removed to reduce immunogenicity, and the obtained acellular matrix was hybridized with chitosan to obtain composite hydrogels. A series of tests revealed that the main components of the composite hydrogels were collagen and polysaccharides and that the hydrogels had good mechanical properties, with the ability to simulate normal nucleus pulposus tissue to a certain extent (Zhang et al. 2022).

Growth factors are known to be involved in the regulation of the proliferation and differentiation of seed cells. In intervertebral disc tissue engineering, commonly used growth factors include basic fibroblast growth factor (BFGF), transforming growth factor (TGF-β1 and TGF-β3), and insulin-like growth factor (IGF-1) (Yoon 2005; Han et al. 2022; Qian et al. 2023). Among the many cytokines, GDF-5 has the most significant effect on promoting the proliferation of nucleus pulposus cells and improving their vitality (Feng et al. 2015). GDF-5 has this effect because it can not only conduct kinase signals to serine threonine receptors to promote differentiation into nucleus pulposus-like cells but can also activate surface proteins to directly increase the viability of directed differentiation into nucleus pulposus-like cells (Guo et al. 2021). However, IDD is a continuous and slow process. The half-life of protein molecules in the body is relatively short, and they easily diffuse after injection. Repeated intradiscal injections are needed, and repeated injections greatly increase the risk of IDD (Skotland et al. 2022). Drug-loaded microspheres synthesized by PLGA can be inhaled or injected, are capable of sustained controlled release, have good biocompatibility, and have no toxic side effects (Shive and Anderson 1997; Hodgkinson et al. 2019; Xiao et al. 2023). In this study, PLGA microspheres were used as carriers to load GDF-5 into the abovementioned composite hydrogels. The microspheres were encapsulated in the hydrogels; therefore, the release of the microspheres was accompanied by the degradation of the hydrogels for further sustained release, and the release of the microspheres was delayed by this physical combination. Testing revealed that GDF-5 was released slowly into the composite hydrogels, with the release efficiency plateauing around the 10-15th day, a finding that was basically consistent with the degradation trend of the composite hydrogels.

To further assess the biocompatibility of composite hydrogels with GDF5-loaded microspheres with stem cells, NPSCs and BMSCs were cultured with or without GDF5 microspheres in two types of composite hydrogel release solutions. Based on the expression levels of the chondrogenic genes ACAN and COL2A1, the composite hydrogels with GDF5-loaded microspheres significantly increased the chondrogenic level of stem cells, and the chondrogenic ability of NPSCs was significantly stronger than that of BMSCs. Further experiments revealed that there was no significant difference in the proliferation levels of NPSCs and BMSCs in the two composite hydrogels loaded or not loaded with GDF5 microspheres. However, with the prolongation of culture time, NPSCs secreted more COL2A1 than did BMSCs. Subsequent animal experiments also showed that although different composite hydrogels loaded with stem cells can alleviate IDD to varying degrees, the composite hydrogels with GDF5-loaded microspheres loaded with NPSCs had the best effect on IDD. We believe that the reason for the above results is that, first, the natural extracellular matrix is the environment where the cells are originally located, and it contains abundant biological factors that can regulate cell proliferation and differentiation. The mechanical properties and biocompatibility can be further improved through the addition of chitosan (Hensley et al. 2018; Norbertczak et al. 2020). Second, NPSCs have tissue specificity, and it is easier for NPSCs to produce the corresponding tissue differentiation effect when the abovementioned composite hydrogels are loaded with NPSCs (Liu et al. 2020).

## Conclusions

In summary, the DNPM/chitosan composite hydrogels prepared in this study can simulate the extracellular matrix of the nucleus pulposus to a certain extent, and the hydrogels can slowly release GDF5 to promote the differentiation of NPSCs into nucleus pulposus-like cells and effectively prevent IDD.

## Data Availability

The datasets used and/or analyzed in the current study are available from the corresponding author on reasonable request.

## Abbreviations

IDD: Intervertebral disc degeneration
GDF5: growth/differentiation factor 5
NPSCs: Nucleus pulposus stem cells
BMSCs: bone marrow mesenchymal stem cells
IPSCs: induced pluripotent stem cells
DNPM: decellularized nucleus pulposus matrix
ACAN: aggrecan
COL2A1: type II collagen

## References

[CR6] Borrelli C, Buckley CT (2020). Injectable disc-derived ECM hydrogel functionalised with chondroitin sulfate for intervertebral disc regeneration. Acta Biomater.

[CR15] Choy AT, Chan BP (2015). A structurally and functionally Biomimetic Biphasic Scaffold for intervertebral disc tissue Engineering. PLoS ONE.

[CR16] Du Y, Li J, Tang X, Liu Y, Bian G, Shi J, Zhang Y, Zhao B, Zhao H, Sui K, et al. The Thermosensitive Injectable Celecoxib-Loaded Chitosan Hydrogel for repairing postoperative intervertebral disc defect. Front Bioeng Biotechnol. 2022;10:876157. 10.3389/fbioe.2022.876157 PMC9274121 35837544

[CR4] Farshad-Amacker NA, Hughes A, Herzog RJ, Seifert B, Farshad M: The intervertebral disc, the endplates and the vertebral bone marrow as a unit in the process of degeneration. 2017, 27(6):2507-2520.5. Walker MH, Anderson DG: Molecular basis of intervertebral disc degeneration. *Spine J* 2004, 4(6 Suppl):158S-166S. 10.1007/s00330-016-4584-z 27709276

[CR23] Feng C, Liu H, Yang Y, Huang B, Zhou Y (2015). Growth and differentiation factor-5 contributes to the structural and functional maintenance of the intervertebral disc. Cell Physiol Biochem.

[CR11] Fontana G, See E, Pandit A (2015). Current trends in biologics delivery to restore intervertebral disc anabolism. Adv Drug Deliv Rev.

[CR1] Fritsch CG, Ferreira PH, Lung T, McLachlan AJ, Ferreira ML. The smallest worthwhile change on function from a self-management intervention for non-persistent low back pain. Eur Spine J 2023. 10.1007/s00586-023-07633-4 37314579

[CR24] Guo S, Cui L, Xiao C, Wang C, Zhu B, Liu X, Li Y, Liu X, Wang D, Li S (2021). The mechanisms and functions of GDF-5 in intervertebral disc degeneration. Orthop Surg.

[CR21] Han F, Yu Q, Chu G, Li J, Zhu Z, Tu Z, Liu C, Zhang W, Zhao R, Mao H (2022). Multifunctional Nanofibrous scaffolds with Angle-Ply microstructure and Co-delivery Capacity promote partial repair and total replacement of intervertebral disc. Adv Healthc Mater.

[CR29] Hensley A, Rames J, Casler V, Rood C, Walters J, Fernandez C, Gill S, Mercuri JJ (2018). Decellularization and characterization of a whole intervertebral disk xenograft scaffold. J Biomed Mater Res A.

[CR27] Hodgkinson T, Stening JZ, White LJ, Shakesheff KM, Hoyland JA, Richardson SM (2019). Microparticles for controlled growth differentiation factor 6 delivery to direct adipose stem cell-based nucleus pulposus regeneration. J Tissue Eng Regen Med.

[CR9] Kamatani T, Hagizawa H, Yarimitsu S, Morioka M, Koyamatsu S, Sugimoto M, Kodama J, Yamane J, Ishiguro H, Shichino S (2022). Human iPS cell-derived cartilaginous tissue spatially and functionally replaces nucleus pulposus. Biomaterials.

[CR31] Liu Y, Li Y, Nan LP, Wang F, Zhou SF, Feng XM, Liu H, Zhang L (2020). Insights of stem cell-based endogenous repair of intervertebral disc degeneration. World J Stem Cells.

[CR2] Lorio MP, Beall DP, Calodney AK, Lewandrowski KU, Block JE, Mekhail N. Defining the patient with lumbar Discogenic Pain: real-world implications for diagnosis and effective clinical management. J Pers Med 2023, 13(5). 10.3390/jpm13050821 PMC10224560 37240991

[CR14] Luo L, Gong J, Wang Z, Liu Y, Cao J, Qin J, Zuo R, Zhang H, Wang S, Zhao P (2022). Injectable cartilage matrix hydrogel loaded with cartilage endplate stem cells engineered to release exosomes for non-invasive treatment of intervertebral disc degeneration. Bioact Mater.

[CR3] Malandrino A, Lacroix D, Hellmich C, Ito K, Ferguson SJ, Noailly J (2014). The role of endplate poromechanical properties on the nutrient availability in the intervertebral disc. Osteoarthritis Cartilage.

[CR7] Mercuri JJ, Gill SS, Simionescu DT (2011). Novel tissue-derived biomimetic scaffold for regenerating the human nucleus pulposus. J Biomed Mater Res A.

[CR30] Norbertczak HT, Ingham E, Fermor HL, Wilcox RK (2020). Decellularized intervertebral discs: a potential replacement for Degenerate Human discs. Tissue Eng Part C Methods.

[CR22] Qian H, He L, Ye Z, Wei Z, Ao J (2023). Decellularized matrix for repairing intervertebral disc degeneration: fabrication methods, applications and animal models. Mater Today Bio.

[CR18] Richardson SM, Hughes N, Hunt JA, Freemont AJ, Hoyland JA (2008). Human mesenchymal stem cell differentiation to NP-like cells in chitosan-glycerophosphate hydrogels. Biomaterials.

[CR17] Roughley P, Hoemann C, DesRosiers E, Mwale F, Antoniou J, Alini M (2006). The potential of Chitosan-based gels containing intervertebral disc cells for nucleus pulposus supplementation. Biomaterials.

[CR8] Shi M, Zhao Y, Sun Y, Xin D, Xu W, Zhou B (2021). Therapeutic effect of co-culture of rat bone marrow mesenchymal stem cells and degenerated nucleus pulposus cells on intervertebral disc degeneration. Spine J.

[CR26] Shive MS, Anderson JM (1997). Biodegradation and biocompatibility of PLA and PLGA microspheres. Adv Drug Deliv Rev.

[CR25] Skotland T, Iversen TG, Llorente A, Sandvig K (2022). Biodistribution, pharmacokinetics and excretion studies of intravenously injected nanoparticles and extracellular vesicles: possibilities and challenges. Adv Drug Deliv Rev.

[CR10] Soufi KH, Castillo JA, Rogdriguez FY, DeMesa CJ, Ebinu JO. Potential role for Stem Cell Regenerative Therapy as a treatment for degenerative disc Disease and Low Back Pain: a systematic review. Int J Mol Sci. 2023;24(10). 10.3390/ijms24108893 PMC10219191 37240236

[CR32] Walker MH, Anderson DG. Molecular basis of intervertebral disc degeneration. Spine J. 2004;4(6 Suppl):158S–166S. 10.1016/j.spinee.2004.07.010 15541661

[CR28] Xiao L, Gao D, Zhang Y, Liu C, Yin Z (2023). Codelivery of TGF-beta1 and anti-mir-141 by PLGA microspheres inhibits progression of intervertebral disc degeneration. J Orthop Surg Res.

[CR20] Yoon ST (2005). Molecular therapy of the intervertebral disc. Spine J.

[CR5] Yurube T, Takeoka Y, Kanda Y, Kuroda R, Kakutani K (2023). Intervertebral disc cell fate during aging and degeneration: apoptosis, senescence, and autophagy. N Am Spine Soc J.

[CR13] Zhang S, Liu W, Wang P, Hu B, Lv X, Chen S, Wang B, Shao Z (2021). Activation of HSP70 impedes tert-butyl hydroperoxide (t-BHP)-induced apoptosis and senescence of human nucleus pulposus stem cells via inhibiting the JNK/c-Jun pathway. Mol Cell Biochem.

[CR19] Zhang Y, Li Y, Liu C, Shang X. Decellularized Nucleus Pulposus Matrix/Chitosan Hybrid Hydrogels for Nucleus Pulposus tissue Engineering. Global Spine J 2022:21925682221135768. 10.1177/21925682221135768 PMC11289557 36330701

[CR12] Zhou L, Xu J, Schwab A, Tong W, Xu J, Zheng L, Li Y, Li Z, Xu S, Chen Z (2023). Engineered biochemical cues of regenerative biomaterials to enhance endogenous stem/progenitor cells (ESPCs)-mediated articular cartilage repair. Bioact Mater.

